# N-butyl cyanoacrylate glue application in prostate artery embolization for benign prostatic hyperplasia: a systematic review of safety and efficacy

**DOI:** 10.1186/s42155-025-00616-0

**Published:** 2025-11-04

**Authors:** Nawaf Salah Ayad Mohamed, Eman El Khatib, Almamoon I. Justaniah, Mohamed E. M. Fouad, Romaric Loffroy

**Affiliations:** 1 College of Medicine, AlMaarefa University, Riyadh, Saudi Arabia; 2 Emirates Health Services, Sharjah, United Arab Emirates; 3 Department of Interventional Radiology, King Faisal Specialist Hospital and Research Center, Jeddah, Saudi Arabia; 4 Department of Vascular and Interventional Radiology, François-Mitterrand University Hospital, Dijon, France

**Keywords:** Prostate artery embolization, N-butyl cyanoacrylate, Benign prostatic hyperplasia, Embolic agents, Interventional radiology

## Abstract

**Background:**

Benign prostatic hyperplasia (BPH) is the most common urological disorder in older males, often treated with prostate artery embolization (PAE) to alleviate lower urinary tract symptoms. While traditional embolic materials like microspheres are common, issues such as symptom recurrence and non-target embolization remain. This systematic review evaluates the safety and effectiveness of n-butyl cyanoacrylate (NBCA) glue as an alternative embolic agent for PAE.

**Materials and methods:**

A thorough search was performed across databases including PubMed, ScienceDirect, Cochrane, Google Scholar, Scopus, and MEDLINE. Studies were included if they assessed NBCA glue for PAE in BPH patients. Exclusions were made for reviews, non-English articles, conference abstracts, and studies not using glue or ethiodized oil mixtures. The Methodological Index for Non-Randomized Studies criteria was used to assess bias risk, and due to varied outcome measures, a narrative synthesis was conducted.

**Results:**

Six studies involving 667 patients met the inclusion criteria. The age in mean ± SD across studies ranged from 67.5 ± 7.8 to 72.6 ± 10.5 with most patients presenting with moderate to severe BPH unresponsive to medication. NBCA glue-based procedures showed high technical success rates and shorter procedure times. International Prostate Symptom Score improvement was reported in 83–94% of patients across all studies with associated quality of life significantly enhanced in up to 94% of patients as well. Prostate volume reduction ranged from 11% to 40.5%, depending on follow-up duration. PSA levels and medication use decreased, and erectile function was mostly preserved, though results varied. Minor complications like groin hematomas and post-embolization syndrome occurred in 4–22% of patients, with no major adverse events reported.

**Conclusion:**

This review assesses NBCA-based glue as a potential embolic agent in PAE for BPH. The evidence suggests promising short-term outcomes with a favorable safety profile, though findings remain preliminary due to small sample sizes and short follow-up. Larger multicenter randomized trials are therefore needed to validate these results and guide clinical practice.

**Graphical Abstract:**

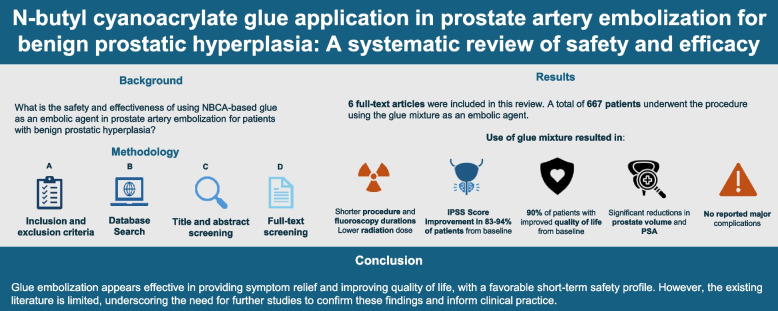

**Supplementary Information:**

The online version contains supplementary material available at 10.1186/s42155-025-00616-0.

## Introduction

Benign prostatic hyperplasia (BPH), a non-cancerous enlargement of the prostate gland, is the most common urological condition among older men, and frequently presents with lower urinary tract symptoms (LUTS) [1, 2]. Treatment varies based upon symptom severity which may include medical and surgical interventions [3, 4]. However, due to adverse effects and poor responses to medical treatments, along with potential surgical complications, alternative procedures like prostate artery embolization (PAE) have emerged. PAE uses fluoroscopic guidance to block prostatic arteries, reducing blood flow to the prostate and causing ischemic necrosis to relieve symptoms [5, 6]. PAE has demonstrated short-term efficacy and quality-of-life improvements, including preservation of sexual function, but long-term outcomes remain under investigation [7–10]. During PAE, various particle-based embolizing agents are used after catheterizing the feeding prostatic arteries, including polyvinyl alcohol (PVA) microspheres, trisacryl gelatin microspheres (Embospheres of 100–500 μm), and polyethylene glycol (PEGM) microspheres. No single embolic agent is definitively superior; the choice depends on the operator’s preference [11, 12]. However, recurrence rates of 23–58.1% and risks of non-target embolization highlight limitations in current embolic agents. This is thought to occur due to the recanalization of previously embolized arteries or prolonged procedures that can reopen existing anastomoses, indicating a need for improved embolic agents [13–15]. Consequently, recent studies explored in this review have assessed the use of n-butyl cyanoacrylate (NBCA) glue as a potential alternative and long-lasting embolic agent. The primary aim of this review is to evaluate the safety and efficacy of NBCA-based glue in PAE among patients with BPH.

## Materials and methods

### Protocol registration

This systematic review was not prospectively registered on PROSPERO but followed PRISMA 2020 guidelines for systematic reviews [16].

### Research question

Primary Research Question: What is the safety and effectiveness of using glue as an embolic agent in prostate artery embolization for patients with benign prostatic hyperplasia?

*Population*: Adult patients with BPH; *Intervention*: PAE using NBCA glue; *Comparison*: Non-glue embolic agents; *Outcome*: Safety (complications), efficacy (clinical success including IPSS, IPSS-QoL, PSA, IIEF5, PV), and technical success.

### Search strategy

The inclusion criteria were primary (observational or experimental) studies published from inception through August 2025, focused on the use of NBCA-based glue in PAE among patients diagnosed with BPH. Exclusion criteria included unpublished studies, editorials, non-English studies, conference abstracts, literature reviews, case reports, or studies not using glue/ethiodized mixture as an embolic agent. The search strategy was then employed with relevant keywords tailored for the databases, including PubMed, Google Scholar, ScienceDirect, Cochrane, Scopus, and MEDLINE:

(“n-butyl cyanoacrylate” OR “NBCA” OR “cyanoacrylate glue” OR “glue” OR “glue embolization”) AND (“prostate artery embolization” OR “prostatic artery embolization” OR “PAE”) AND (“benign prostatic hyperplasia” OR “BPH”).

### Study screening and eligibility

**Fig. 1:**
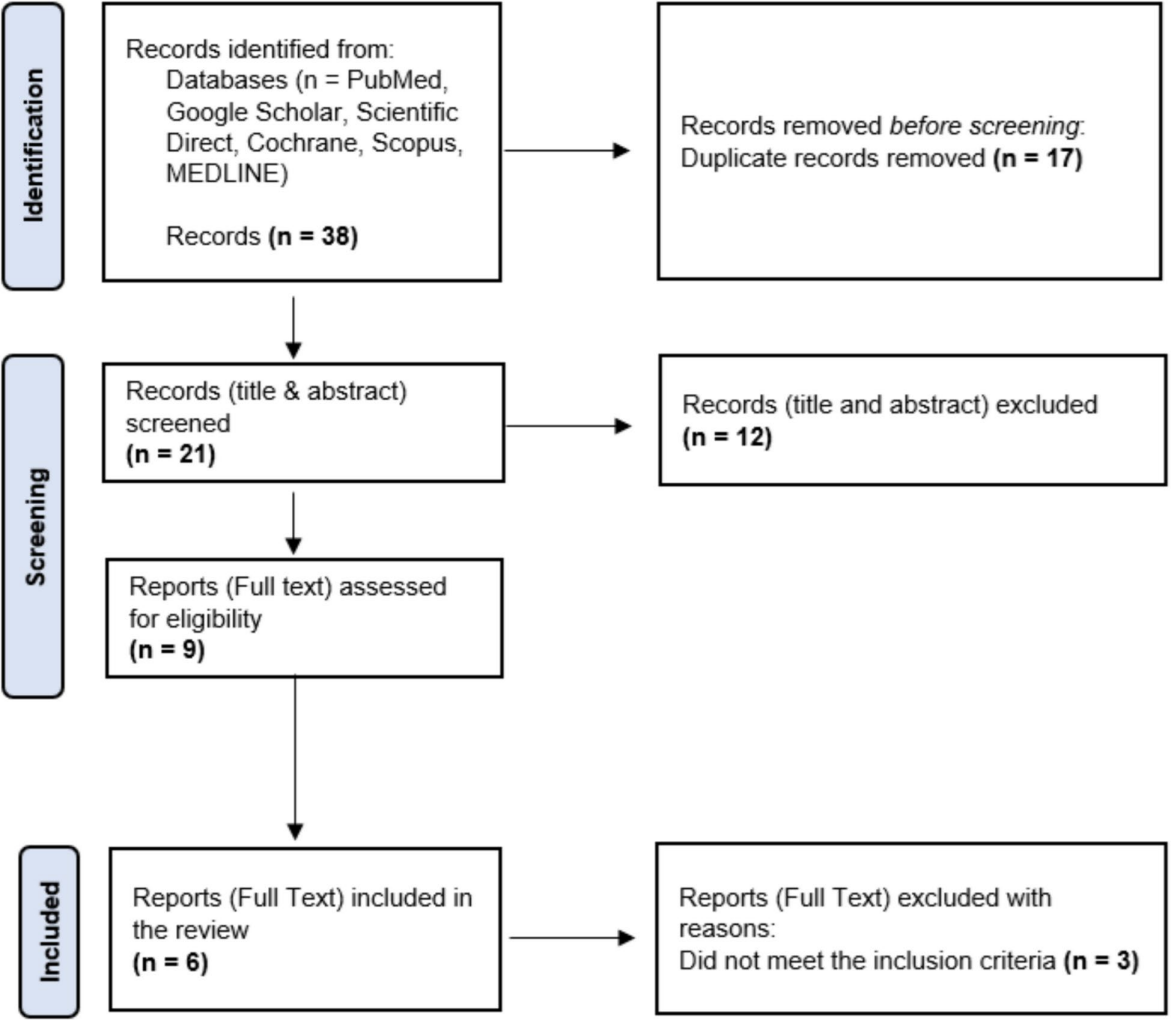
The PRISMA flow diagram

A total of 38 studies were initially collected via Zotero citation software and uploaded into Rayyan, a web-based review site that aids in the screening of studies, where 17 duplicates were then removed [17, 18]. The title and abstract screening of 21 studies was performed by two independent reviewers based on the inclusion criteria. If there was a disagreement between the reviewers, a third reviewer made the final decision. Subsequently, full-text screening was performed on 9 studies by two independent reviewers, ending with a total of 6 selected studies. Figure 1 summarizes the screening process through the use of a PRISMA (Preferred Reporting Items for Systematic Reviews and Meta-Analyses) flow diagram [16].

### Data extraction and synthesis

Data were extracted using a standardized Excel sheet (Supplementary Material). Owing to the limited number of studies and heterogeneity in outcome reporting and follow-up durations, a quantitative meta-analysis was not feasible. Instead, a qualitative narrative synthesis was performed in accordance with PRISMA 2020 as well as the Synthesis Without Meta-analysis (SWiM) guidelines [16, 19]. Studies were grouped by follow-up duration (≤ 3 months, 6 months, 12 months) and by outcome domain (IPSS, IPSS-QoL, PV, PSA, IIEF5, safety). Outcomes were summarized using structured tabulation and reported as ranges rather than pooled means to accommodate heterogeneity in follow-up intervals and study design. This structure enabled consistent comparisons across heterogeneous studies and the progression of PAE treatment effects overtime. Variability in clinical and methodological characteristics—including prostate size, embolization technique, and operator experience—was qualitatively explored to interpret outcome differences.

### Procedural overview

#### Patient selection

Criteria for patient selection were mostly similar among all included studies, as most patients were required to present with LUTS due to BPH. The inclusion criteria generally encompassed the following: patients aged over 50, a duration of LUTS for at least 6 months, a lack of response to standard BPH pharmacotherapy, refusal or contraindications to surgical interventions, an IPSS score exceeding 7, an IPSS-QoL score greater than 2, and a prostate volume exceeding 30 mL. Notably, Hijazi et al. established higher thresholds, requiring an IPSS of 18 points or more and an IPSS-QoL score of 3 or higher instead [20]. Additionally, the exclusion criteria included biopsy-confirmed prostate cancer, active urinary tract infection, severe atherosclerosis, advanced renal impairment, individuals under the age of 50, past prostatic surgery, an IPSS score lower than 7, and a QOL score of 2 or below. Furthermore, Bamshad et al. excluded patients who had embolization performed with secondary microsphere agents or those who sought contralateral embolization after an initial unilateral procedure to avoid potential confounding variables [21].

#### Patient preparation and technical success

**Fig. 2:**
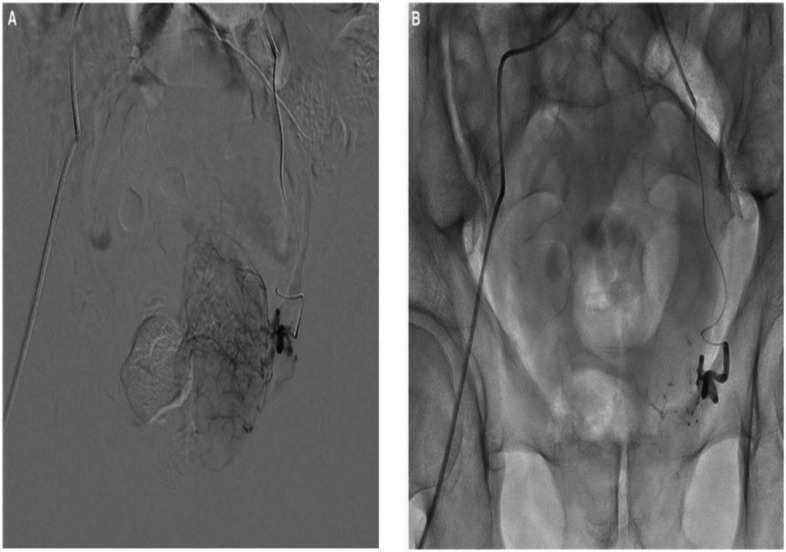
The possible incorporation and utilization of glue/ethiodized mixture in PAE for a patient with BPH. **A** Left prostatic artery angiogram before PAE showing enhancement of the left prostatic lobe. **B** Follow-up angiogram after PAE with a mixture of Glubran^®^2/Lipiodol in a 1:8 ratio showing total occlusion. Adapted from the corresponding author with permission [22]

Patients receive prophylactic antibiotics to reduce infection risk. Technical success is defined as the complete occlusion of at least one artery supplying the prostate, confirmed by arteriography as seen in Fig. 2.

#### Procedure details

Procedures were mostly outpatient, performed at academic or tertiary institutions by one experienced interventional radiologist. One study by Loffroy et al. reported two operators instead [23]. The procedures were performed using either the common femoral or the left radial artery, accordingly. The iliac arteries were selected using a 4- or 5- Fr Cobra or Simmons type 2 catheter or otherwise. Arteriograms were performed using different projections to identify the prostatic artery.

Superselective catheterization of the prostatic arteries was performed using a 2.0–2.7 Fr microcatheter (Progreat; Terumo, Tokyo, Japan) in most studies. Bamshad et al. further detailed the use of a 2.0-F balloon occlusion microcatheter (Sniper; Embolx, Sunnyvale, California), a 2.0-F microcatheter (TruSelect; Boston Scientific, Marlborough, Massachusetts), and 2.4 and 2.8-F microcatheters (Direxion; Boston Scientific) [21].

Routine use of cone-beam CT angiography was used to map arterial anatomy and minimize non-target embolization.

#### Embolization material

A mixture of iodized oil and NBCA-based glue is prepared immediately before injection. The glue/ethiodized mixture is diluted at a ratio of 1:8 to 1:10 (Glue: Ethiodized oil) to optimize flow dynamics and enhance radiopacity.

#### Injection technique

Before embolization, 1 mg of isosorbide dinitrate (Risordan, 10 mg/10 mL vial) was administered intra-arterially on each side to induce vasodilation. Additionally, 10 mL of 5% dextrose solution was flushed through the microcatheter and vascular bed to prevent premature polymerization of the glue and promote adequate distal embolization. The injection was then performed using either a free-flow technique with continuous delivery or a blocked-flow technique for arteries requiring a controlled intermittent delivery of the embolic agent. The liquid nature of NBCA-based glue allows injection in vessels where the microcatheter cannot be advanced when injected in a blocked-flow manner, thus enabling more distal embolization with less reflux [23, 24]. During the injection process, continuous fluoroscopic monitoring is employed, and the procedure is halted when there is clear evidence of substantial reflux (such as occurring over the first 1–2 mm of the microcatheter tip situated in the middle third of the prostatic artery). This method ensures successful embolization while minimizing collateral filling.

#### Record of procedural metrics and follow-up

Procedural metrics such as radiation dose (dose-area product) measured in mGy·cm^2^, fluoroscopy time per minute, and total PAE duration were documented as mean ± SD. Patients were monitored post-procedure and followed up at 1, 3, 6, and 12 months. BPH medications were tapered gradually. Antibiotics and anti-inflammatories were prescribed upon discharge.

### Outcome assessment

#### Efficacy outcomes

Outcomes reflecting the efficacy of the procedure refer to the degree of improvement in symptom severity and quality of life post-PAE. Thus, various data points (red as mean ± SD) were collected at the baseline and post-procedure for comparative analysis. Few studies in this review were also comparative by assessing the clinical outcomes of the glue/ethiodized mixture to established embolic agents.

Symptoms were measured using the IPSS scale, which ranges from 0 to 35, with higher scores indicating more severe symptoms. Quality of life was evaluated through the IPSS-QoL score, a single question evaluating the patient’s perceived burden of their urinary condition, asking how they would feel if their symptoms continued throughout their life, on a scale from 0 (“delighted”) to 6 (“terrible”). Clinical success was generally defined by most studies as an IPSS-QoL score of less than 3, accompanied by a decrease of 25% or more in IPSS from the baseline, or an IPSS score below 18 [22]. Primary outcomes included the IPSS and IPSS-QoL parameters. Secondary outcomes encompassed the prostate volume (PV) in milliliters (mL), and prostate-specific antigen (PSA) levels in ng/mL, which may reflect prostatic inflammation and the degree of ischemia that would be imposed post-procedure. The prostate volume was measured using either magnetic resonance imaging or ultrasound in all included studies. Another relevant parameter for secondary outcomes included the International Index of Erectile Function (IIEF5) score, ranging from 0 to 25, with greater scores indicating better erectile function. All 6 included studies reported outcomes using validated clinical metrics (e.g., IPSS, IPSS-QoL, PSA in ng/mL, PV in mL, IIEF5 score). No data transformation or conversion into standardized effect sizes was performed, as these metrics are routinely used and directly comparable across studies [20, 21, 23–26].

#### Safety outcomes

The safety of the procedure was assessed by recording complications and categorizing their severity using the Society of Interventional Radiology (SIR) classification and the Clavien-Dindo grading system by the studies included in this review [20, 21, 23–26]. Complications were classified as minor (SIR A and B; Clavien-Dindo I and II) if manageable with outpatient treatment, or major if they resulted in extended hospitalization, re-admission, or required further surgery.

#### Quality and risk of bias assessment

The quality assessment of the included studies was performed using the Methodological Index for Non-Randomized Studies (MINORS). The MINORS is a validated tool for assessing the methodological quality of non-randomized studies, including cohort, case–control, and comparative observational studies [27]. It consists of 8 items for non-comparative studies and 4 additional items for comparative studies, each scored on a 0–2 scale, with a maximum score of 16 for non-comparative studies and 24 for comparative studies. Two authors independently assessed the quality of the included studies using the MINORS tool, with disagreements resolved through discussion or consultation with a third author.

## Results

### Patient demographics

A total of 667 patients across six studies underwent PAE using a glue/ethiodized mixture. Most patients who underwent the procedure had medically refractory BPH or declined surgery. The age in mean ± SD ranged from 67.5 ± 7.8 to 72.6 ± 10.5 across studies and was commonly presented with diagnosed comorbidities such as diabetes mellitus, hypertension, and/or dyslipidemia. Pre-procedure IPSS (mean ± SD) ranged from 19.6 ± 6.61 to 25.7 ± 4.3, and IPSS-QoL ranged from 4.0 ± 1.1 to 5.1 ± 0.9, indicating moderate to severe symptoms. Prostate gland (PV) volume ranged from 94 ± 39.9 to 134.1 ± 88.4 mL [20, 21, 23–26].

### Study design

Most included studies were retrospective and based in France, China, and the United States. Study durations ranged from 2–4 years, with a target population ranging from 30 to 252 samples. Follow-up durations across the included studies varied from one to twelve-month intervals [20, 21, 23–26]. Table 1 summarizes the characteristics of the included studies.

**Table 1:** Characteristics of the included studies (*N* = 6)

**Included studies**	**Country**	**Journal**	**Study design**	**Sample size**	**Study period**
Loffroy et al. 2021 [23]	France	J. Clin. Med	Retrospective	50	November 2017 to October 2020
Salet et al. 2022 [25]	France	Cardiovasc. Intervent. Radiol	Retrospective	30	September 2017 to July 2020
Hijazi et al. 2023 [20]	China	J. Urol	Prospective	54	July 2019 to June 2021
Loffroy et al. 2024 [24]	France	Diagn. Interv. Imaging	Retrospective	103	September 2018 to January 2023
Bamshad et al. 2024 [21]	United States	J. Vasc. Interv. Radiol	Retrospective	244	June 2022 to May 2024
Loffroy et al. 2025 [26]	France	Eur. Radiol	Retrospective	186	September 2018 to December 2023

### Procedural characteristics

The average procedure duration ranged from 95.0 ± 2.90 to as low as 80.7 ± 22.5 min. Fluoroscopy duration was significantly reduced with the use of glue, ranging from 24.9 ± 11.7 to 27.5 ± 11.3 min (*p* = 0.0002) in comparative studies. Similarly, the average radiation dose ranged from 1144 ± 1021 to 32,495 ± 34,569 u/mGy.cm^2^, with the Kerma area product being significantly lower (*p* = 0.0001) with glue/ethiodized mixtures as well [20, 21, 23–26]. Table 2 provides an overview of the available procedural characteristics among included studies.

**Table 2:** Summary of the procedural characteristics (*N* = 6)

Included studies	Technical success definition	Type of glue	Glue dilution ratio	Injection technique	Radiation dose u/mGy (Mean ± SD)	Fluoroscopy duration min (Mean ± SD)	Total PAE duration min (Mean ± SD)
Loffroy et al. 2021 [23]	Unilateral or bilateral embolization—3 (6%), 47 (94%)	NBCA (Glubran 2, GEM; Viareggio, Italy)	1:8	Free or blocked flow	18,458 ± 16,397	27.5 ± 11.3	95.0 ± 29.0
Salet et al. 2022 [25]	Unilateral or bilateral embolization—2 (6.7%), 28 (93.3%)	NBCA (Glubran 2, GEM, Italy)	1:9	N/A	11,995 ± 6702	25 ± 9.1	80.7 ± 22.5
Hijazi et al. 2023 [20]	Unilateral or bilateral embolization—54 (100%)	NBCA (Glubran 2, GEM; Viareggio, Italy)	1:8	N/A	N/A	N/A	N/A
Loffroy et al. 2024 [24]	Unilateral embolization or bilateral embolization—10 (9.7%), 93 (90.3%)	MS-NBCA mixture (Glubran 2, GEM)	1:8	Free or blocked flow	32,495 ± 34,569	26.4 ± 12.5	91 ± 35
Bamshad et al. 2024 [21]	Bilateral embolization—219 (95%)	NBCA (Trufill; Cerenovus, Miami, Florida)	1:10	N/A	1144 ± 1021	24.9 ± 11.7	N/A
Loffroy et al. 2025 [26]	Bilateral embolization—186 (100%)	NBCA (Glubran 2, GEM; Viareggio, Italy)	1:8	Free or blocked flow	32,378 ± 33,549	32.6 ± 18.5	86 ± 31

### Primary outcomes

IPSS improvement was reported in 83–94% of patients across all studies. Reported mean reductions from baseline ranged from 9.5 points at short-term follow-up (≤ 3 months) to as high as 18.5 points at 6 months, with a sustained improvement of 10 points at 12-month follow-up [20, 25, 26]. Specifically, Loffroy et al. reported improvement in 90% of cases (45/50) at 3-month follow-up, while Salet et al. noted a slightly lower rate of 83% (25/30) at 3-month follow-up [23, 25]. In a separate study, Loffroy et al. observed IPSS improvement in 87.4% of patients (90/103) at 6-month follow-up, while Bamshad et al. reported a similar outcome in 92% (110/119) of patients at 1-month follow-up [21, 24]. Loffroy et al. again observed IPSS improvement in 94.6% of patients (176/186) at 12-month follow-up [26]. Additionally, Hijazi et al. documented an improvement of 10.2 points at three months, and an additional 8.3 points at six months (*p* < 0.0001) [20].

Concerning IPSS-QoL, all studies also reported significant post-procedural improvements, with up to 90% of patients demonstrating clinically meaningful changes from baseline (*p* < 0.05) [20, 21, 23–26]. Notably, a QoL score of less than 3 was achieved in up to 86% of patients at both three and six-month follow-up intervals by Loffroy et al. [23, 24]. Similarly, Bamshad et al. reported a mean QoL enhancement of 2.2 points in 94% of patients after an average follow-up of 7.1 weeks [21]. By univariate analysis, Loffroy et al. in another study observed a significant QoL improvement from baseline on a 12-month follow-up instead (*p* < 0.0001) [26].

Loffroy et al. also observed a marked decrease in the requirement for pharmacological management of LUTS, with 90% of patients no longer requiring medication at a 3-month interval [23]. Salet et al. also reported comparable findings, with 67% of patients discontinuing pharmacotherapy at six months [25]. Another study by Loffroy et al. observed the proportion of patients taking medications decreased to as low as 21% at 12 months [26]. Table 3 summarizes the primary clinical outcomes observed during follow-up after glue/ethiodized mixture embolization in PAE.

**Table 3:** Summary of follow-up primary outcomes (*N* = 6)

Included studies	Follow-up duration	IPSS score (Mean ± SD)	IPSS-QoL (Mean ± SD)
**Loffroy et al. 2021** [23]	Baseline	20.5 ± 6.7	4.9 ± 1.0
	**Follow-up (3 months)**	**9.9 ± 6.8**	**2.2 ± 1.5**
** Salet et al. 2022** [25]	Baseline	19.6 ± 6.61	4.8 ± 1
	**Follow-up (130 ± 91 days)**	**10.1 (Mean)**	**2.4 (Mean)**
** Hijazi et al. 2023** [20]	Baseline	25.7 ± 4.3	4.43 ± 0.2
	**Follow-up (3 months)**	**15.5 ± 2.3**	**2.3 ± 0.4**
	**Follow-up (6 months)**	**7.2 ± 1.09**	**1.5 ± 2.27**
** Loffroy et al. 2024** [24]	Baseline	20.2 ± 6.5	5.1 ± 0.9
	**Follow-up (6 months)**	**8.9 ± 6.2**	**2.1 ± 1.4**
** Bamshad et al. 2024** [21]	Baseline	20.7 ± 6.8	4.0 ± 1.1
	**Follow-up (7.1 weeks)**	**9.5 ± 6.0**	**1.8 ± 1.5**
** Loffroy et al. 2025** [26]	Baseline	20.1 ± 6.4	5.1 ± 0.9
	**Follow-up (12 months)**	**10.0 ± 6.6**	**2.2 ± 1.6**

### Secondary outcomes

All included studies reported a significant PV reduction from baseline on follow-up with varying degrees.

Specifically, Hijazi et al. reported an 11% mean difference from baseline (67.7 ± 8.5 to 56.7 ± 7.9 mL) at 3-month follow-up and a greater 27.5% mean difference (67.7 ± 8.5 to 40.2 ± 5.4 mL) at the 6-month follow-up [20]. Likewise, Loffroy et al. reported a 21% mean difference from baseline (98.3 ± 40.2 to 77.3 ± 30.5) at 3-month follow-up and a greater 40.5% mean difference (119.1 ± 65.7 to 78.6 ± 43.4) at 6-month follow-up [23, 24]. Loffroy et al. also reported a 31.9% mean difference from baseline (118.1 ± 67.4 to 81.1 ± 47.0) at 12-month follow-up [26]. A larger baseline PV was also shown to be independently associated with a greater IPSS improvement at follow-up as reported by Loffroy et al. [23, 24].

In terms of mean PSA levels, a significant decline from baseline was noted by Loffroy et al. as there was a drop of 1.8 ng/mL (28.1%) at 3-month follow-up and 1.2 ng/mL (25%) at 6-month follow-up, respectively [23, 24]. However, no drop was noted by Loffroy et al. at 12-month follow-up with a 2% increase from baseline instead [26].

IIEF5 outcomes were mixed, as three studies found no significant improvement. Loffroy et al. specifically found that the mean IIEF5 score was reduced by 0.4 points at three months (15.8 ± 7.9) compared to baseline (16.2 ± 7.5) (*p* = 0.078) in one study, while it was reduced by 0.5 points (15.3 ± 6.8) compared to baseline (15.8 ± 6.8) (*p* = 0.078) in another study [23, 24]. However, Hijazi et al. reported a 10-point increase at six-month follow-up (19.3 ± 1.3) compared to baseline (9.4 ± 1.5) (*p* < 0.0001) instead [20]. Another study by Loffroy et al. observed a 0.4 point increase (16.2 ± 7.2) compared to baseline (15.8 ± 6.9) (*p* = 0.8140) at 12-month follow-up [26].

Clinical heterogeneity was observed among studies due to differences in baseline prostate volume, embolization technique, and follow-up intervals. Methodological heterogeneity was also evident, primarily because most studies were retrospective and non-comparative. These factors were qualitatively considered when interpreting the narrative ranges reported, rather than combining data into pooled averages. Table 4 summarizes the secondary clinical outcomes observed during follow-up after glue/ethiodized mixture embolization in PAE.

**Table 4:** Summary of follow-up secondary outcomes (*N* = 6)

**Included studies**	**Follow-up duration**	**PSA level (ng/mL)** **(Mean ± SD)**	**IIEF5** **(Mean ± SD)**	**PV (mL)** **(Mean ± SD)**
** Loffroy et al. 2021** [23]	Baseline	6.4 ± 3.7	16.2 ± 7.5	98.3 ± 40.2
	**Follow-up (3 months)**	**4.6 ± 3.0**	**15.8 ± 7.9**	**77.3 ± 30.5**
** Salet et al. 2022** [25]	Baseline	N/A	N/A	94 ± 39.9
	**Follow-up (130 ± 91 days)**	**N/A**	**N/A**	**78.6 (Mean)**
** Hijazi et al. 2023** [20]	Baseline	N/A	9.4 ± 1.5	67.7 ± 8.5
	**Follow-up (3 months)**	**N/A**	**14.5 ± 1.1**	**56.7 ± 7.9**
	**Follow-up (6 months)**	**N/A**	**19.3 ± 1.3**	**40.2 ± 5.4**
** Loffroy et al. 2024** [24]	Baseline	4.8 ± 4.2	15.8 ± 6.8	119.1 ± 65.7
	**Follow-up (6 months)**	**3.6 ± 3.2**	**15.3 ± 6.8**	**78.6 ± 43.4**
** Bamshad et al. 2024** [21]	Baseline	N/A	N/A	134.1 ± 88.4
	**Follow-up (7.1 weeks)**	**N/A**	**N/A**	**113 ± 69**
** Loffroy et al. 2025** [26]	Baseline	4.8 ± 4.2	15.8 ± 6.9	118.1 ± 67.4
	**Follow-up (12 months)**	**4.9 ± 3.3**	**16.2 ± 7.2**	**81.1 ± 47.0**

### Safety outcomes

Only minor complications (4–22%) were reported, with no major adverse events [20, 21, 23–26]. The most common complications were groin hematomas (1.9–10%), postembolization syndrome (3.3–10.7%), and transient erectile dysfunction (1–8%), all of which were resolved with conservative management. Other complications included pain (1.0–3.3%), superficial mucosal glans penis necrosis (1.0–3.3%), and urinary tract infection (1.0–2.0%), all of which were mild and self-limited.

### Quality and risk of bias assessment

The MINORS tool was used to assess the quality of six non-randomized studies included in this review. The total score ranged from 9 to 22, with a mean score of 13.0. The items with the lowest scores across studies were the unbiased assessment of study endpoints (score of 0 in all studies) and the prospective collection of data and calculation of study size (score of 0 in all but one study), reflecting the retrospective design of most included studies. Conversely, the highest-scoring items were a clearly stated aim, appropriate endpoints, and an adequate follow-up period, each consistently scoring 2 in all studies. Table 5 summarizes the detailed MINORS scoring for each included study.

**Table 5:** Methodological index for non-randomized studies (*N* = 6)

	Item	Loffroy et al. 2021 [23]	Salet et al. 2022 [25]	Hijazi et al. 2023 [20]	Loffroy et al. 2024 [24]	Bamshad et al. 2024 [21]	Loffroy et al. 2025 [26]
**Criteria for non-randomized non-comparative studies**	**A clearly stated** **aim**	2	2	2	2	2	2
	**Inclusion of** **consecutive** **patients**	2	2	2	2	2	2
	**Prospective** **collection of** **data**	0	0	2	0	0	0
	**Endpoints** **appropriate to** **the aim of the** **study**	2	2	2	2	2	2
	**Unbiased** **assessment of** **the study** **endpoint**	0	0	0	0	0	0
	**Follow-up** **period** **appropriate to** **the aim of the** **study**	2	2	2	2	1	2
	**Loss to follow-** **up less than** **5%**	2	2	2	2	1	1
	**Prospective** **calculation of** **the study size**	0	0	2	0	0	0
**Additional criteria for comparative studies**	**An adequate control group**	-	2	2	-	-	-
	**Contemporary groups**	-	2	2	-	-	-
	**Baseline equivalence of groups**	-	2	2	-	-	-
	**Adequate statistical analyses**	-	2	2	-	-	-
**Total score**		**10/16**	**18/24**	**22/24**	**10/16**	**9/16**	**9/16**

## Discussion

This review assessed the safety and efficacy of NBCA-based glue in PAE for BPH. The included studies varied in baseline prostate volume, embolization technique, and follow-up. Most procedures were conducted at high-volume academic centers, limiting the generalizability of the findings. Across six studies, NBCA-based glue consistently demonstrated short-term improvements in symptom relief as reflected by reductions in IPSS and QoL metrics without major complications. However, these findings should be interpreted cautiously, given the predominance of retrospective data, small sample sizes, and limited follow-up durations. Additionally, NBCA glue use was associated with shorter procedural and fluoroscopy times, reducing radiation exposure.

### NBCA efficacy

Clinical success was defined as an IPSS-QoL score < 3 and ≥ 25% IPSS reduction. NBCA-based glue achieved an IPSS improvement in 83–94% of patients, with QoL scores falling by 2 points, remaining under 3 in all studies [20, 21, 23–26].

In alignment with these findings, Sapoval et al. conducted a prospective study involving 405 patients who underwent PAE for bothersome LUTS using 100–300 μm or 300–500 μm Embosphere microspheres and reported a comparable improvement in IPSS of 12.4 points at 3-month follow-up compared to baseline (21.8 ± 6.6 to 9.3 ± 6.6) [28]. However, IPSS improvement was 11.1 points at 12 months (21.8 ± 6.6 to 10.6 ± 7.5) and 10.6 points at 24 months (21.8 ± 6.6 to 11.2 ± 7.9), indicating potential symptom recurrence [28].

Similarly, Ray et al. conducted a prospective study with 305 patients, reporting an IPSS improvement of 10.9 points at 12-month follow-up [29]. Additionally, a comparative study of 300–500 μm microspheres and non-spherical PVAs by Hwang et al. involving nine patients reported a smaller IPSS improvement of 9.8 points at 10.1-month follow-up on average (24.6 ± 9.7 to 14.7 ± 9.4) [30].

Although glue-based embolization showed symptom relief advantages in some studies, other agents occasionally outperformed it depending on prostate size and baseline symptom severity. A prospective trial conducted by de Assis et al. on 35 patients with prostate volumes larger than 90 g using 300–500 μm microspheres showed a higher IPSS improvement of 15.6 points at 3-month follow-up [31]. Similarly, Torres et al. investigated 138 patients in a prospective study comparing microspheres of different sizes and demonstrated larger IPSS improvements across all groups. Mean improvements in IPSS were 13.02 points with 100–300 μm microspheres (23.0 ± 5.62 to 9.98 ± 6.67), 14.76 points with 300–500 μm microspheres (23.0 ± 5.15 to 8.24 ± 6.57), and 14.1 points with 100–300 μm microspheres followed by a 300–500 μm microspheres combination (24.2 ± 4.89 to 10.1 ± 5.90) [32]. Differences may stem from a higher baseline symptom severity and longer follow-up durations, which may have allowed more time for symptom improvement.

It should be noted that the evidence base for PAE as a viable treatment option for BPH is emerging with different embolic microspheres, as similar improvements have also been reported with both trisacryl gelatin microspheres and PEGM [13, 33]. This review aligns with these findings by presenting comparable efficacy outcomes with the use of the NBCA glue/ethiodized mixture.

In terms of PV mL, NBCA-based embolization achieved reductions up to 40.5 mL at 6-month or so follow-up [20, 21, 23–26]. This mirrors results with PVAs, PEGM, and microspheres, which have shown PV decreases between 12.7 and 40.4 mL [13, 28–33]. Although the lowest reported mean baseline volume was 94 ± 39.9 mL in this review, studies have demonstrated feasibility and technical success in prostates as small as 50–80 mL, suggesting potential applicability to smaller gland sizes [34, 35].

Regarding mean PSA levels, a noticeable decline was observed with one available study reporting a drop of 1.8 ng/mL (28.1%) at 3-month follow-up, 1.2 ng/mL (25%) at 6-month follow-up while another study reported no reduction at 12-month follow-up [23, 24, 26]. These findings mostly align with embosphere microspheres used in a previous study, highlighting a drop of 1.4 ng/mL at 12-month follow-up [32]. However, some studies suggest otherwise, with 100–500-um embosphere microspheres producing a greater reduction of 3.1 to 4.7 ng/mL (58.8%) at three-month follow-up [13, 31]. In addition, PEGM reported a greater reduction of 6.0 ng/mL (58.6%) at 12-month follow-up [33]. Generally, these significant findings with embolic agents may not necessarily correlate with symptomatic improvement, as it is expected for elderly men to have a relatively enlarged prostate along with higher PSA levels. Symptom relief does not always correlate with PV or PSA changes, requiring cautious interpretation.

IIEF5 scores varied. Three studies reported a slight insignificant decrease ranging from 0.4 to 0.5 points at 3 and 6-month follow-up or a 0.4 increase at 12-month follow-up instead [23, 24, 26]. In contrast, another study observed a significant increase of 9.9 points at 6-month follow-up, with no significant differences observed between the glue/ethiodized mixture and PVAs [20]. This particular improvement not only aligned with previous studies but also surpassed the aforementioned embolic agents, which typically reported an increase of 1 to 4.1 points at 12-month follow-up [28, 32]. The inconsistencies could be influenced by a multitude of factors including the baseline IIEF5 score, follow-up timing, and psychological factors such as reduced anxiety from symptom relief. Overall, NBCA glue appears effective in preserving or improving erectile function.

### Other surgical procedures

In evaluating clinical success, transurethral resection of the prostate (TURP) was associated with greater improvements in IPSS compared to embolic agents as reported by Ray et al. Specifically, TURP produced a 15.2-point improvement from baseline at 12-month follow-up. Additionally, embolic agents did not consistently outperform TURP in this department [29].

In terms of PV mL, most embolic agents demonstrated superior outcomes compared to TURP at the 3-month follow-up in one study, achieving a mean difference of 29.1 mL from baseline compared to a change of only 6.9 mL with TURP, although this was based on incomplete data for extended follow-up [29].

Regarding IIEF5, all embolic agents exhibited better performance than TURP, which only recorded a slight increase of 0.2 points at the 12-month follow-up. However, this finding is to be expected as accidental damage to collateral structures within the procedure can cause a higher rate of retrograde ejaculation (47.5 to 70%) and reduction of ejaculatory function. In addition, most studies would report varying results on the effect of TURP on erectile function or IIEF5, providing little to no significant differences [29, 36, 37]. Overall, the findings suggest that the glue/ethiodized mixture appears comparable to other embolics and less invasive than TURP, supporting its potential use as a first-line treatment.

### NBCA safety

On one hand, the glue/ethiodized mixture demonstrated a minor complication rate of 4% to 22% which is lower than that reported with TURP (1.6 to 63.9%) [28]. Reported complications included groin hematomas and transient erectile dysfunction, while no major events (e.g., UTIs, bladder ischemia) were observed [20, 21, 23–26]. The term “glans penis necrosis” may sound alarming, but three studies have shown it to be superficial and self-limiting, resolving within three weeks or with conservative treatment, thus not requiring surgery. Therefore, it is appropriately classified as a minor complication by SIR and Clavien-Dindo criteria [23, 24, 26].

Additionally, there was no significant correlation between the embolic techniques used in PAE (microsphere versus glue embolization) and the occurrence of immediate or delayed complications [25]. Compared to embosphere microspheres and PVA (≥ 11.1% minor complications, 1.2–2.9% major), NBCA glue appears favorable [28, 31]. PEGM also demonstrated a safe profile (13.6% minor complications, no major events) [33].

Most complications reported were also similar to the aforementioned embolic agents, with non-glue/ethiodized mixture agents reporting self-limited urinary infection–related symptoms as the most common [13, 28–33]. This suggests glue/ethiodized mixtures may offer a favorable safety profile among current embolic options.

Moreover, the procedure duration was notably shorter with the glue/ethiodized mixture across all included studies. One study reported a longer procedure duration (112 ± 42.1 min), fluoroscopy duration (42.4 ± 20.3 min), and radiation dose (65,143 ± 120,316 uGy·m^2^) with 300–500 μm microspheres (Embogold) in comparison [25]. Another study reported a much longer procedure duration (135.13 ± 76.24 min), fluoroscopy duration (41.82 ± 26.57 min), and radiation dose (203.2 ± 38.6 Gy/cm^2^) with PEGM instead [33].

The lower procedure along with the fluoroscopy durations seen with glue/ethiodized mixture contributes to lower patient and operator exposure to radiation dose, potentially minimizing the risk of complications and hazards [23–25]. Consequently, these procedural advantages may make glue preferable to microparticles in certain settings. However, outcomes still depend on operator expertise and experience. While microspheres may remain the standard use due to extensive research in comparison, NBCA-based glue shows promise in reducing radiation exposure and procedure duration.

### NBCA advantages

When mixed with ethiodized oil, NBCA glue achieves efficient vascular occlusion with minimal complications [38]. This homogeneous mixture is designed to achieve distal embolization while occluding blood flow to the prostate effectively. The ethiodized oil also imparts radiopacity to the mixture, allowing real-time fluoroscopic visualization during injection, unlike microparticles, which cannot be directly traced during delivery. Second, the liquid nature of the NBCA–Lipiodol mixture enables embolization of small-caliber or low-flow vessels that may be inaccessible to microcatheters [39]. This approach allows both distal and proximal occlusion, which may reduce recanalization and symptom recurrence.

### Research limitations

Initially, a significant number of studies employed retrospective designs, potentially affecting the reliability of outcomes due to missing data and small sample sizes. Additionally, some studies reported relatively short follow-up durations—as brief as one month—which may not adequately capture long-term outcomes. This is particularly relevant given that late recanalization has been documented following PAE when microparticles are used. The small number of studies as well as the heterogeneity in techniques and outcomes limits generalizability. Few studies directly compared glue to other embolics, and objective measures such as uroflowmetry were rarely reported, which restricts the ability to comprehensively assess the treatment’s clinical impact. Additionally, bilateral embolization was more common than unilateral, precluding comparative analysis. Potential bias may also stem from operator experience and procedural consistency, as most procedures were performed by a small group of interventional radiologists with specific expertise in glue/ethiodized mixture embolization or microsphere embolization. This may have conferred an advantage in procedural execution and influenced the reported outcomes. Alongside the limitations present in the studies included, our synthesis methodology possesses its own inherent restrictions as meta-analysis was considered impractical, leading to a narrative summary of findings organized by follow-up duration and outcome domain.

### Areas for future research

Future studies should prioritize robust, multicenter randomized controlled trials with larger cohorts to reduce the methodological biases associated with retrospective studies. More studies should extend the follow-up intervals—preferably six months or longer—to further accurately assess the long-term efficacy of glue embolization, particularly regarding symptom relief and recanalization rates, which may not be apparent within shorter observation periods. Furthermore, studies should include a comparative evaluation of various embolic agents, including glue/ethiodized mixtures, PVA particles, and other materials, to better elucidate their relative safety and efficacy profiles. The inclusion of objective functional measures, such as uroflowmetry and uroflow dynamics, can also be essential to provide a more comprehensive objective assessment of treatment clinical outcomes. Addressing these gaps will contribute to a more complete understanding of the clinical value of glue embolization and support its integration into evidence-based PAE practice.

## Conclusion

This review evaluates the use of NBCA glue as an embolic agent in PAE for BPH. The available evidence suggests encouraging short-term outcomes with a favorable safety profile. However, the current literature is limited by small cohorts, heterogeneous designs, and short follow-up durations, underscoring the need for larger multicenter randomized controlled trials with longer follow-up periods to validate these findings and guide future clinical practice.

## Supplementary Information

Supplementary Material 1.

## Data Availability

Not applicable.

## Abbreviations

BPH: Benign prostatic hyperplasia
PAE: Prostate artery embolization
LUTS: Lower urinary tract symptoms
IPSS: International Prostate Symptom Score
PRISMA: Preferred Reporting Items for Systematic Reviews and Meta-Analyses
QoL: Quality of life
PV: Prostate volume
IPSS-QoL: International Prostate Symptom Score Quality of Life
NBCA: N-butyl cyanoacrylate
IIEF5: International Index of Erectile Function
SIR: Society of Interventional Radiology
PVA: Polyvinyl alcohol microspheres
PEGM: Polyethylene glycol microspheres
TURP: Transurethral resection of the prostate
mL: Milliliters
MINORS: Methodological Index for Non-Randomized Studies
